# Brodie’s abscess following percutaneous fixation of distal radius fracture in a child

**DOI:** 10.1007/s11751-016-0249-3

**Published:** 2016-03-16

**Authors:** Karthig Rajakulendran, Natasha E. Picardo, Ibraheim El-Daly, Rami Hussein

**Affiliations:** Basildon and Thurrock University Hospitals NHS Foundation Trust, Nethermayne, Essex SS16 5NL UK

**Keywords:** Brodie’s abscess, Kirschner wires, K-wire, Infection, Subacute osteomyelitis

## Abstract

We report the case of a Brodie’s abscess presenting five and a half years following closed reduction and percutaneous pinning of a distal radius fracture. The index surgery was complicated by a pin site infection that was treated successfully with antibiotics. The patient represented with forearm pain years later, and radiological investigations revealed a Brodie’s abscess in the distal radius at the site of the previous Kirschner wires. The Brodie’s abscess was managed through surgical curettage and antibiotics. Staphylococcus aureus and diphtheroid organisms were cultured from the intraoperative specimens. A Brodie’s abscess is a form of localised subacute osteomyelitis, which usually occurs in the metaphysis of long bones and can mimic malignancy. Previous trauma or surgery has been implicated as predisposing factors. We have only identified one previously reported case of Brodie’s abscess following percutaneous pinning. Ours is the first reported case in an adolescent. The aim of this paper is to raise awareness of this rare complication and review the current literature.

## Introduction

Percutaneous pinning using Kirschner wires (K-wires) is a common method used to manage unstable distal radius fractures. We report the case of a teenager who developed a Brodie’s abscess several years after undergoing a closed reduction and percutaneous pinning of a distal radius fracture. A review of the literature surrounding Brodie’s abscess is presented. Due to the morbidity associated with K-wiring, this should be reserved for unstable fractures and full aseptic techniques used.

## Case report

**Fig. 1:**
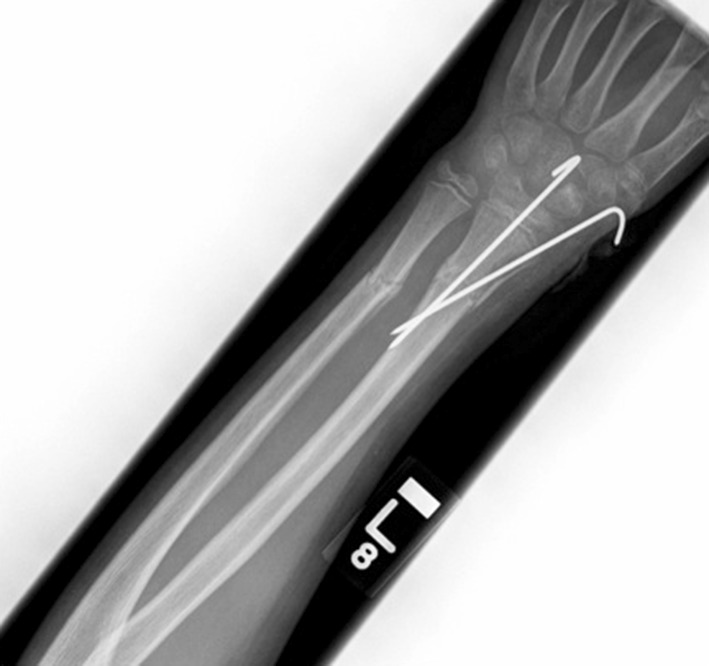
AP radiograph of the *left* forearm showing percutaneous K-wire fixation of a distal radius fracture

A 16-year-old, right-hand-dominant school boy attended the accident and emergency department with a 2-week history of increasing left forearm pain and swelling. There was no significant trauma, and the patient denied any systemic symptoms. He had sustained a closed fracture of the left distal radius five and a half years previously, whilst playing rugby. The fracture was managed at the time of injury with closed reduction and stabilisation using two percutaneous K-wires (Fig. 1), and one dose of antibiotics was given on induction. The patient had developed an infection at the radial styloid pin site 1 month following the original surgery. The K-wires were removed at the time, and he was given a 10-day course of oral flucloxacillin and placed into a full cast. Wound swabs grew *Staphylococcus aureus*. The infection resolved, but he developed an area of excess granulation tissue around the infected pin site that was treated with silver nitrate.

**Fig. 2:**
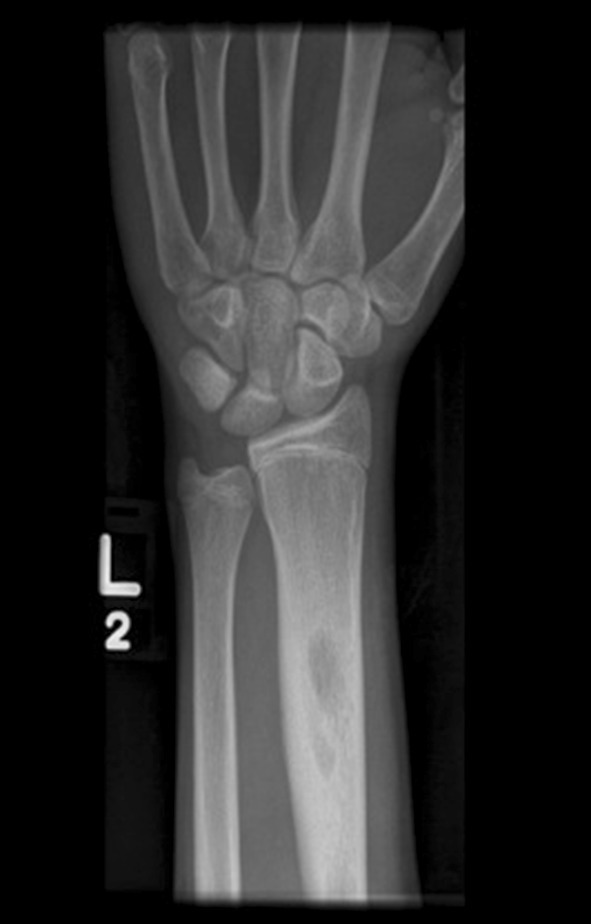
AP radiograph of the *left* distal radius showing an intramedullary lytic lesion

**Fig. 3:**
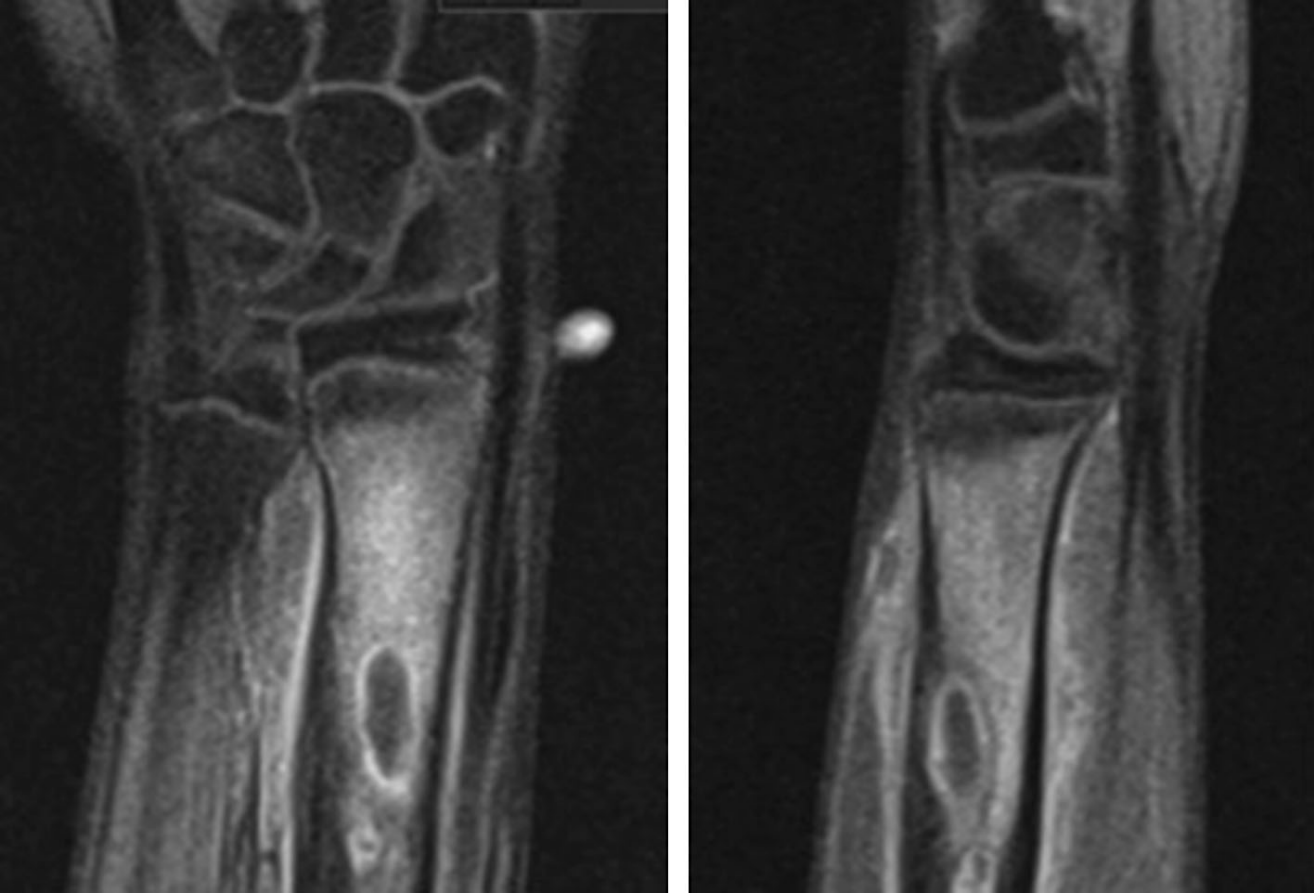
MRI scan of the *left* distal radius showing cystic lesions in the distal radius with surrounding oedema and an area of cortical discontinuity

**Fig. 4:**
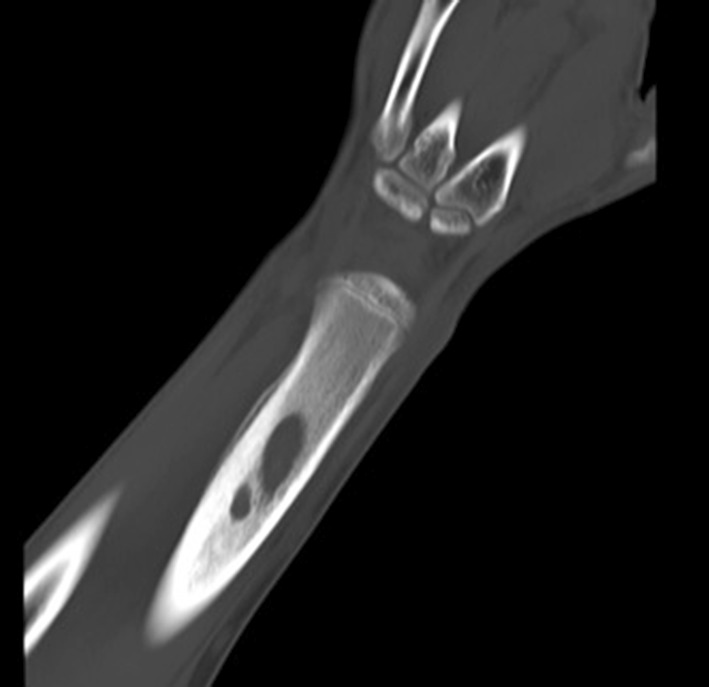
CT scan of the *left* distal radius confirming the lucent area with cortical thickening

The patient was asymptomatic until the representation five and a half years later. He had no co-morbidities and took no regular medication. Examination revealed a firm tender swelling on the dorsal aspect of the forearm with no surrounding erythema or skin changes. He demonstrated a full range of movement in the wrist and elbow joints with no irritability. There was no associated neurovascular deficit and he was afebrile. Blood tests showed a raised C-reactive protein of 10 and a normal white blood count. A plain radiograph of the forearm revealed a lytic lesion in the distal radial diaphysis, with an ill-defined edge, wide zone of transition and evidence of cortical expansion and periosteal reaction (Fig. 2). The patient underwent a contrast-enhanced magnetic resonance imaging (MRI) scan that demonstrated two discrete fluid-filled lesions in the distal radius (measuring 2.0 cm × 0.9 cm and 1.1 cm × 0.5 cm), with surrounding bone marrow and soft tissue oedema (Fig. 3). A computer tomography (CT) scan confirmed the lucent area, with cortical thickening and an area of cortical discontinuity (Fig. 4). The scans were discussed with the regional bone and soft tissue sarcoma unit. The opinion was that the images were consistent with a Brodie’s abscess in the radius, with inflammatory changes extending across the growth plate and a small dorsal periosteal abscess at the site of the break.

The patient was taken to theatre for exploration and curettage of the lesion, under fluoroscopic guidance. A dorsal skin incision was made centred over the cortical breach in the distal radius. The defect was identified and an osteotome used to widen the cavity mouth and open the second smaller cavity. The cavities contained no pus, but were found to have a lining of granulation tissue. Multiple samples were sent for microbiology and histology. The cavities were curetted until healthy bleeding bone was exposed and then irrigated with chlorhexidine and hydrogen peroxide. They were then packed with a Betadine-soaked wick, and the wound was left open. A second look and washout were performed 48 h later. The tissues appeared healthy, with no signs of residual infection, and the wound was closed. The intraoperative specimens grew *Staphylococcus aureus* and diphtheroid organisms. Following discussion with the microbiologist, the patient was treated with intravenous flucloxacillin 2 g QDS for 5 days, followed by oral flucloxacillin 1 g for 3 months. In addition, oral fusidic acid 500 mg TDS was administered for the same duration.

**Fig. 5:**
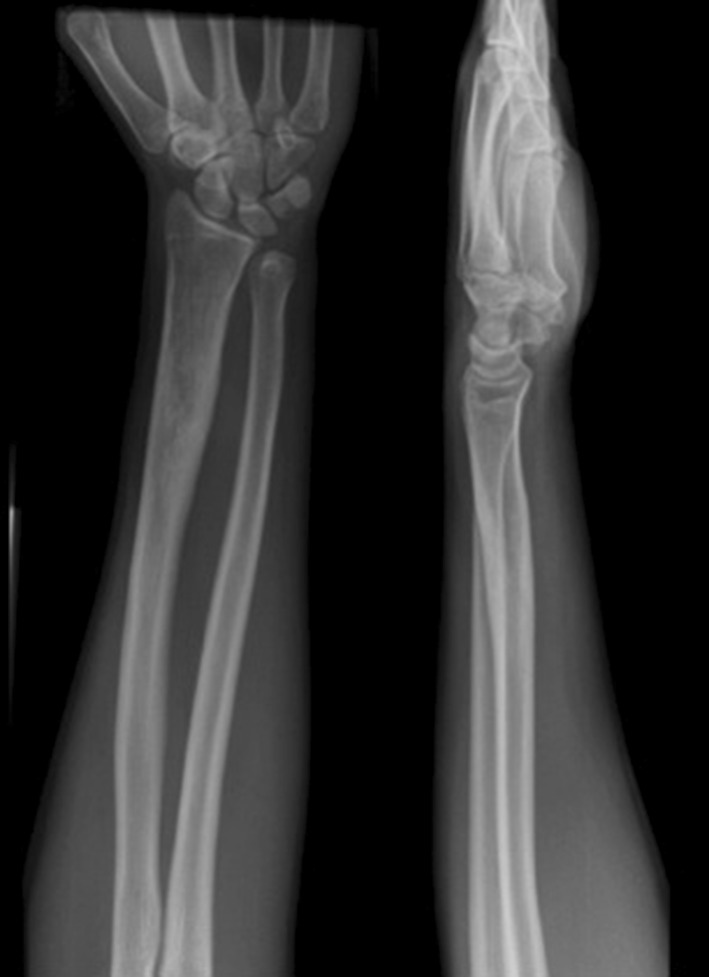
Radiographs 1 year after curettage of Brodie’s abscess

**Fig. 6:**
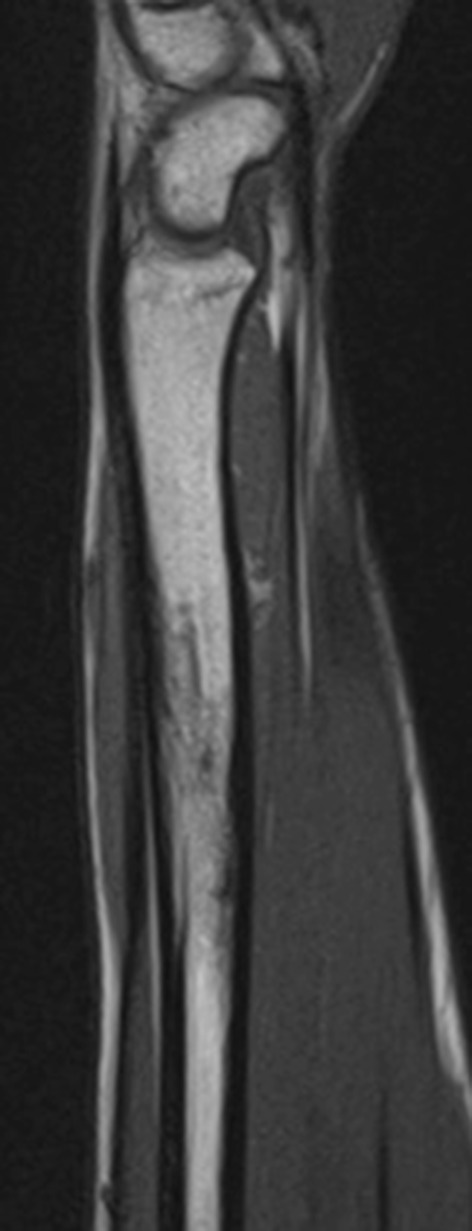
MRI scan performed 1 year after curettage of Brodie’s abscess

At the 3-month follow-up, the patient reported no post-operative complications and only minor discomfort on performing strenuous activity. He demonstrated a full painless range of movement in the left arm, and inflammatory markers were normal. At 12 months, blood test results were again unremarkable and the patient was completely asymptomatic. Radiographs and an MRI scan were repeated (Figs. 5, 6).

## Discussion

Distal radius metaphyseal fractures are the most common childhood fractures (20.2 %) [1]. Fixation with percutaneous K-wires is recommended in patients who carry high risk of reduction loss after closed treatment in order to prevent displacement and preserve rotational capacity of the forearm. Such patients include those with complete initial fracture displacement and those with ipsilateral distal ulnar fractures [2]. Although used for more than 100 years in fracture management, there is a paucity of evidence regarding timing of K-wire removal and antibiotic prophylaxis. Botte et al. [3] have shown in adults that the incidence of infection increases with the length of time the K-wires are left in situ. In the paediatric patient with a distal radius fracture, the K-wire is usually removed at 3–4 weeks [4]. In our experience, one intraoperative dose of antibiotics is given.

Whilst pin tract infections are a well-documented complication, with a reported incidence of 2–21 % [5, 6], the formation of a Brodie’s abscess following the insertion of metal wire is a rare phenomenon. A Brodie’s abscess is a form of localised subacute osteomyelitis. Patients typically present with pain, swelling and warmth. In some cases, the onset is insidious, whilst others are asymptomatic and discovered as an incidental finding, or following relatively minor trauma [7]. The lesions are most often found in the diaphysis or metaphysis of tubular bone, commonly in the lower extremities, particularly the tibia [8]. Isolated case reports have documented their appearance in less common sites, such as the calcaneum and cuboid bone [9]. In the paediatric population, it is thought that Brodie’s abscess often manifests in the vascular-rich metaphyseal region of long bones following haematogenous spread during a bacteraemia or remote infection [7, 10]. They can also arise directly, secondary to previous trauma or surgery in the affected bone [11]. *Staphylococcus aureus* is the most common organism isolated, with Streptococcus viridans and Pseudomona also being reported. However, in 20–25 % of cases no organism can be isolated [12].

The history and appearance of a Brodie’s abscess can present a diagnostic challenge. The differential diagnosis includes benign and malignant processes, such as aneurysmal bone cysts, fibrous dysplasia, non-ossifying fibroma, osteoid osteoma, eosinophilic granuloma, giant cell tumours, osteoblastoma and Ewing sarcoma [13]. Blood analysis may reveal elevated inflammatory markers, but are often unremarkable. Imaging, however, can often distinguish a Brodie’s abscess from other pathology. Plain radiographs typically demonstrate a well-defined intramedullary lucent lesion with sclerotic margins [8]. The presence of cortical thickening and periosteal reaction is variable. Bone scans may show an increase in radioisotope uptake. MRI offers high sensitivity, and a “target appearance” with four layers seen on T1-weighted imaging is reported as characteristic, but not pathognomonic. This comprises a hypointense central abscess cavity, an inner ring of intermediate signal intensity, an outer ring of hypointensity and a peripheral hypointense halo [14]. The outer ring represents the area of reactive osteosclerosis, whilst the inner rings correspond to a layer of granulation tissue in the abscess wall, and shows enhancement following the addition of contrast. Surrounding bone marrow and soft tissue oedema are frequently observed. Scintigraphy can be performed in cases where multi-focal infection is suspected. In cases where malignancy cannot be excluded from imaging and laboratory investigation, a biopsy is advocated.

The management of Brodie’s abscess consists of aggressive surgical debridement and curettage of the cavity, with the use of bone graft in large defects, typically more than 3 cm [15]. Antibiotics are given in conjunction, although the duration of treatment is variable, with recommendations ranging from 6 to 12 weeks [12, 16]. Stephens and MacAuley [15] reported a recurrence in eight of the 21 cases they managed with curettage and antibiotics. Their case series spanned 21 years, and they had no consistency in antibiotic choice or duration (2–8 weeks). However, they noted that all patients with an initial ESR greater than 44 mm/h had a recurrence and therefore advocated a more aggressive surgical decompression and longer duration of antibiotics for these patients. Olasinde et al. [12] reported excellent two-year outcomes with no recurrence in a series of 20 patients treated with curettage, autologous bone graft harvested from the anterior iliac crest and a 6-week course of antibiotics. Shih et al. [17] reported good outcome following curettage and the implantation of local gentamicin-polymethylmethacrylate beads. The use of percutaneous drainage of a Brodie’s abscess has also recently been described [18].

A literature search identified only one other reported case of Brodie’s abscess 4 years after percutaneous pinning in an adult [19]. In this case, however, the patient presented later, and after an initial period of pain, swelling and erythema had developed a recurrently discharging abscess which had been incised and drained on multiple occasions. Our case is the first report of a Brodie’s abscess as a long-term complication following percutaneous pinning in a paediatric patient.

The case highlights the importance of asepsis and adhering to operating theatre principles. This case also highlights the importance of removing K-wires at the shortest safe interval if they are protruding through the skin. Some surgeons bury the wires, and this should be considered if wires are to be left in longer. This case highlights the need for early detection and treatment of pin track infections, to reduce the risk of progression to deep infection. The late manifestation illustrates the insidious nature of the pathology and the need to maintain a high index of suspicion when patients develop symptoms at a later stage. Furthermore, it reinforces the importance of assessing stability in distal radius fractures, as the introduction of k-wires can be associated with significant morbidity and should only be used in unstable configurations.
